# Associations of Ultra‐Processed Foods and Executive Function in Children With Obesity and Their Parents: A Cross‐Sectional Study

**DOI:** 10.1111/ijpo.70143

**Published:** 2026-08-16

**Authors:** Ingrid Rivera‐Iñiguez, David R. Strong, Kyung E. Rhee, Dawn M. Eichen, Kerri N. Boutelle

**Affiliations:** ^1^ Department of Pediatrics University of California San Diego San Diego California USA; ^2^ Herbert Wertheim School of Public Health and Human Longevity Science University of California San Diego San Diego California USA; ^3^ Department of Psychiatry University of California San Diego San Diego California USA

**Keywords:** childhood obesity, executive function, ultra‐processed foods

## Abstract

**Background:**

Ultra‐processed foods (UPFs) are linked to obesity and cognitive decline, but evidence on their association with executive function (EF) in children is limited.

**Objective:**

To evaluate the UPFs‐EF relationship in parent–child dyads with obesity seeking treatment.

**Methods:**

Secondary analysis of the FRESH trial (NCT01197443) conducted at the University of California, San Diego. A total of 141 Parent–child dyads; parent age = 43 years (6.6), BMI = 32 (6.4); child age = 9.9 years (1.3), BMIz = 2.0 (0.3); 31% Hispanic, 44% White, 25% other completed anthropometric, digit span backward (DSB), stop signal task (SST) and Wisconsin Card Sorting Test (WCST), and dietary assessments. Linear regression models evaluated UPFs‐EF associations, controlling for covariates.

**Results:**

Parent–child associations were observed for energy *β* = 0.15, *p* = 0.024; total UPFs *β* = 0.20, *p* = 0.018; savoury snacks *β* = 0.28, *p* = 0.018; ultra‐processed (UP) meat, *β* = 0.60, *p* < 0.001; and sweetened beverages *β* = 0.24, *p* = 0.023. Most UPFs‐EF associations were null. In children, UP meat predicted lower DSB scores (*β* = −0.104, 95% CI [−0.197, −0.013], *p* = 0.013), and in parents, sweetened beverages predicted failed SST (*β* = 0.606, 95% CI [0.018, 1.210], *p* = 0.044).

**Conclusions:**

There were a few associations between UPFs intake and EF; however, most were nonsignificant. Future research should involve community samples and children across the weight spectrum.

**Abbreviations**
BMI: body mass index
BMIz: standardized body mass index *z*‐score
CHEAR: eating and activity research
CVD: cardiovascular disease
dAGEs: dietary‐derived advanced glycation end products
DSB: digit span backward
DSBss: digit span backward scaled score
EF: executive function
FRESH: family, responsibility, education, support and health trial
NCC: Nutrition Coordinating Center
NDS‐R: Nutrition Data Systems for Research
RT: reaction time
SST: stop signal task
TMAO: trimethylamine‐oxide
UPFs: ultra‐processed foods
WAIS‐IV: Wechsler Adult Intelligence Scale Fourth Edition
WCST: Wisconsin Card Sorting Test
WISC‐IV: Wechsler Intelligence Scale for Children Fourth Edition

## 1 Introduction

In the United States (U.S.), the prevalence of overweight and obesity among children and adolescents is approximately 21.1% [1]. The health consequences associated with obesity in children encompass psychological, physical and metabolic conditions [2]. Children with obesity are at a heightened risk of developing chronic conditions in adulthood, such as high blood pressure, high cholesterol, Type 2 diabetes and sleep apnea [3]. It is crucial to recognize that childhood obesity is a multifactorial issue, and dietary intake is one of the most significant modifiable risk factors [3].

The widespread availability of ultra‐processed foods (UPFs) has magnified the caloric contribution of fats and sugars in today's diet [4]. UPFs are hyperpalatable foods produced through industrial processes that result in products with increased energy density, fats, sugars, sodium and food additives such as preservatives and artificial dyes [4]. The consumption of these foods has escalated, comprising approximately 58% of energy intake among adults and around 67% among children in the U.S. [5, 6]. The excessive intake of UPFs has been associated with multiple adverse health implications, such as a greater risk of obesity, Type 2 diabetes, depression, heart disease and a rise in all‐cause mortality [7]. In children, high consumption of these foods is associated with a higher standardised body mass index *z*‐score (BMIz) [8], waist circumference, fat mass, fasting plasma glucose and lower HDL cholesterol. Proposed biological mechanisms explaining the adverse health effects of UPFs include increased oxidative stress and disturbances in the endocrine system and gut microbiota [7].

Childhood represents a crucial developmental phase for brain maturation, particularly with respect to executive function (EF). EF encompasses a range of cognitive skills related to purposeful and goal‐directed behaviour, including working memory, inhibitory control and cognitive flexibility [9]. Research indicates that both adults and children with obesity show deficits in EF, notably in areas such as inhibitory control, working memory and cognitive flexibility [10, 11]. Additionally, research shows that obesity can adversely influence the development of brain structures involved in EF [12].

Studies indicate that adults and children with lower EF experience significant challenges in regulating portion sizes, resisting cravings, overeating when not hungry, and indulging in high‐calorie foods [13, 14]. Among children with obesity, poorer cognitive flexibility and decision‐making are associated with greater fat intake; lower working memory is associated with decreased planning for healthy foods, and lower cognitive flexibility is associated with suboptimal adherence to dietary recommendations [15, 16].

In addition to obesity‐related effects, UPFs may independently contribute to impaired EF through multiple mechanisms, including glucose and insulin abnormalities and overstimulation of dopamine pathways that affect impulse control and decision‐making immediately after consumption [7]. Additionally, UPFs promote gut dysbiosis leading to neuroinflammation [17]. Furthermore, food additives and compounds from processing and packaging can disrupt the endocrine system, promote oxidative stress and impair neuroplasticity [7, 18]. Studies show that UPFs are associated with poorer EF in older adults and a higher risk of cognitive decline [19, 20]. Similarly, a greater intake of sugar‐sweetened beverages and processed snack foods was negatively associated with EF in adolescents and children [21]. In children, frequent consumption of candy and sweet baked goods was linked to a lower verbal comprehension index among 4–7‐year‐old [22]. Moreover, maternal consumption of UPFs exceeding 28.9% of daily energy was associated with a reduced verbal skill score in children aged 4–5 years [23]. In contrast, adolescents (15–18 years old) with low cognitive performance did not demonstrate significant differences in UPFs consumption compared with those with medium‐high cognitive performance [24]. However, evidence examining the relationship between UPFs and EF in children is limited.

The few studies linking dietary patterns and EF have not simultaneously examined UPFs consumption and EF among children with obesity, while also including their parents, especially using the current gold‐standard NOVA classification [25]. Previous research has examined children or adults separately, limiting understanding of how intergenerational dietary patterns, shared household food environments and parental modeling of eating behaviors shape children's dietary intake and related cognitive outcomes. In addition, little is known about the relationships in families seeking obesity treatment, where family‐level influences may be especially relevant.

Thus, the purpose of this study was to evaluate the relationship between UPFs and the three core domains of EF (working memory, inhibition and cognitive flexibility) among parent–child dyads prior to enrolling in an intensive lifestyle intervention for weight management [26]. We hypothesised that increased consumption of UPFs would be associated with poorer EF in both parents and children. Additionally, we hypothesised that parental UPFs consumption would be highly related to the child UPFs consumption.

## 2 Methods

### 2.1 Study Design and Participants

This cross‐sectional analysis used baseline data from 141 parent–child dyads seeking treatment for child weight management who participated in the Family, Responsibility, Education, Support and Health (FRESH) trial (NCT01197443) at the University of California San Diego (UC San Diego) Center for Healthy Eating and Activity Research (CHEAR) (July 2011–July 2015). Recruitment methods, measures, interventions and outcomes have been described elsewhere [26, 27]. Inclusion criteria were: Children 8–12 years old with a BMI between the 85th and 99.9th percentile for age and sex, one biological parent in the household with a BMI ≥ 25 kg/m^2^ (i.e., overweight), parent with a reading level of at least the 5th grade in English, and willingness to participate in study visits over 2 years. Exclusion criteria included serious current physical disease (e.g., diabetes), any medical condition that would make physical activity unsafe, current substance use disorder, family food restrictions or allergies, religious or ethnic practices that limit the foods available in the home, any medical or psychological problems that could make adherence to the study protocol difficult or dangerous (e.g., purging), and concurrent enrolment in another weight management program. All measures for the current analysis were collected during baseline, prior to randomisation. The UC San Diego and Rady Children's Hospital Institutional Review Boards approved this study. All parents completed informed written consent, and children provided written assent prior to study enrolment. All measures were completed by both parent and child unless indicated.

### 2.2 Measures

#### 2.2.1 Dietary Intake

Both children and parents reported their usual dietary intake by completing three 24‐h dietary recalls over a span of three non‐consecutive days (including 2 weekdays and 1 weekend day) via telephone interviews conducted by experienced dietitians using the validated USDA's Automated Multiple‐Pass Method [28]. Children self‐reported their dietary intake with parental assistance. Dietary recalls were conducted separately from EF testing. The Nutrition Data Systems for Research (NDS‐R) software developed by the Nutrition Coordinating Center (NCC), University of Minnesota, Minneapolis, MN [29], was used to calculate dietary intake by averaging the three recalls.

Food group servings were estimated utilising the NCC Food Group Serving Count System [29]. Food groups were categorised according to the level of processing, using the NOVA Food classification system [25], Table S1. NOVA is the most widely used food classification system employed in epidemiological studies [30]. This system classifies foods into four distinct categories: Category 1: unprocessed or minimally processed foods: no processing or mostly using processes to make single foods more durable, accessible, convenient, palatable or safe; Category 2: processed culinary ingredients: the extraction and purification of components of single whole foods; Category 3: processed foods: products created by the addition of salt, oil, sugar or other ingredients from Categories 1 and 2 through methods such as canning, bottling, non‐alcoholic fermentation; and Category 4 UPFs: processing of a mixture of ingredients from other categories to yield durable, accessible, convenient, palatable ready‐to‐eat or to‐heat products that most likely will be consumed as snacks or will replace dishes usually prepared at home. The percentage of energy obtained from UPFs in relation to total energy intake was calculated.

#### 2.2.2 Executive Function

##### 2.2.2.1 Digit Span

To assess working memory, the Digit Span subtest from the Wechsler Intelligence Scale for Children, Fourth Edition (WISC‐IV) for children [31] and the Wechsler Adult Intelligence Scale Fourth Edition (WAIS‐IV) for parents [32] was utilised. The examiner read a set of numbers aloud, and the participant had to repeat strings of numbers that became progressively longer, both in the same order and in reverse order. The Digit Span Backward scaled score (DSBss) was used in this analysis, with lower scores indicating poorer working memory capabilities.

##### 2.2.2.2 Stop Signal Task

The stop signal task (SST) was used to evaluate inhibitory control [33]. The test included 288 trials delivered in 6 blocks with 72 total stop trials. Parent–child dyads pressed the left arrow key on the keyboard as quickly as possible when an ‘X’ appeared on the screen or pressed the down arrow key when an ‘O’ appeared and withheld their response if they heard a tone (stop trial). In a practice session, a mean reaction time (RT) was established for each participant and then individualised RT to be either hard (delivered at RT, or 100 or 200 ms less than RT) or easy (delivered 300, 400 or 500 ms less than RT). For each stop signal, the percentage of trials that failed to inhibit was calculated and then averaged to create a single score. A higher score indicates an increased number of trials with unsuccessful inhibition.

##### 2.2.2.3 Wisconsin Card Sorting Test

The Wisconsin Card Sorting Test (WCST) evaluated cognitive flexibility using a computerised version [34]. Participants were presented with a card and instructed to categorise it into one of four available category cards distinguished by colour, shape and number. Participants were provided feedback as to whether they chose right or wrong, forcing them to use trial and error to determine how to sort the cards. Throughout the testing process, the categorisation criteria were covertly altered, unbeknownst to the participants. Errors were considered perseverative when the participant failed to adjust their response based on the provided feedback and continued to adhere to an incorrect sorting rule. To measure cognitive flexibility, the count of perseverative errors, represented by *T*‐scores, was used, with higher *T*‐scores signifying a greater number of errors and poorer performance.

#### 2.2.3 Anthropometrics

Anthropometric measurements, including body weight and height, were obtained by trained staff. Body weight was measured in kilograms in duplicate using a Tanita Digital Scale (model WB‐110A), with participants wearing light clothing and no shoes, and recorded to the nearest 0.1 kg; the average of the two values was used for analysis. Height was measured in centimeters in triplicate using a Seca stadiometer (model 222) while participants stood barefoot with heels, buttocks and upper back against the vertical backboard. The head was positioned in the Frankfort horizontal plane, and measurements were recorded to the nearest 0.1 cm. The average of the three values was used for analysis. Body weight and height were used to calculate the Body Mass Index (BMI) (kg/m^2^) for adults, and age‐adjusted BMI *z*‐score (BMIz) was computed for children using the Center for Disease Control growth charts [35].

#### 2.2.4 Physical Activity

Parents and children wore a GT1M Actigraph accelerometer (ActiGraph Corp) for a minimum of four out of 7 days to evaluate their physical activity levels. Data were extracted, processed and scored with the ActiLife software, version 6.11 (ActiGraph Corp), which provided transformed summaries aggregated across 30‐second epochs. Activity estimates were categorised into intensity levels of physical activity using calibration thresholds previously validated for adults [36] and children [37]. The mean daily estimates of moderate and vigorous‐intensity physical activity were considered in the present analysis.

#### 2.2.5 Demographics

Demographic data pertaining to parents and children, including age, sex, race, ethnicity educational attainment and household income, were collected through a demographic survey completed by the parents concerning both themselves and their child. A detailed list of all socio‐demographic items is provided as a Supporting Information.

#### 2.2.6 Statistical Analysis

UPF intake was operationalised as the percentage of total energy intake derived from UPFs. Pearson correlation coefficients were calculated to assess the association between parental and child UPF intake.

To further assess this association while accounting for potential confounding variables, multiple linear regression models were used, controlling for age, sex, race, income, education, parental BMI or children's BMIz and physical activity, as these factors have been consistently associated with dietary intake [38, 39]. To evaluate the association between UPF‐EF, multiple linear regression models were used, with total UPF and UPF subcategories modelled as continuous variables. All models were adjusted for age, sex, race, income, education, parental BMI or children's BMIz and physical activity, as these factors have been previously associated with EF [9, 40, 41, 42]. All models were conducted separately for each UPF subcategory, including sweetened beverages, sugars and desserts, savoury snacks, meats, grains, fats, dairy and artificially sweetened beverages. Our analyses were conducted at the family level, examining each parent–child dyad. Data for parents and children were analysed separately, with each model incorporating a single observation per family. The analyses were conducted using baseline data only. All participants in the analytic sample had complete data for the variables of interest; therefore, no missing‐data imputation was required. All study variables were analysed using the R software, version 4.3.2, with the Tidyverse, jtools, ggplot2 packages.

## 3 Results

### 3.1 Parent and Child Demographics and Clinical Characteristics

Table 1 presents the demographic, anthropometric, physical activity and EF data. The majority of the participants were female (88% of parents and 67% of children). The overall sample was composed of 44% White individuals and 30.5% Hispanic individuals. Approximately 51% of parents held a college degree.

**TABLE 1:** Parent and child demographics and clinical characteristics.

Characteristic	Parent *n* = 141	Child *n* = 141
	Mean (SD)	Mean (SD)
Age (years)	43 (6.6)	9.9 (1.3)
BMI and Child's BMIz	32 (6.4)	2.0 (0.34)
Sex % (*n*)		
Female	125 (88)	95 (67)
Male	16 (12)	46 (33)
Race % (*n*)		
Hispanic	43 (31.0)	43 (30)
Non‐Hispanic White	62 (44.0)	62 (44)
Non‐Hispanic Other	34 (24.0)	34 (24)
Unreported	2 (1.0)	2 (1.0)
Parent education % (*n*)		
Graduate degree	43 (30)	—
College	51 (36)	—
Partial college	28 (20)	—
High school graduate	10 (7)	—
Partial high school	2 (1)	—
Unreported	7 (5)	—
Physical activity, min/d	33.6 (20.4)	179.4 (47.2)
Executive function		
DSB scaled score	7.7 (2.3), 2–14, IQR 3	10 (2.7), 4–16, IQR 4
SST % trials of failed inhibition	45 (14.0), 17–83, IQR 19.4	48 (17.0), 0–132, IQR 19
WCST perseverative errors (*T*‐scores)	47 (8.7), 20–80, IQR 7	53 (10.0), 27–76, IQR 15

*Note:* Continuous variables are presented as mean and standard deviation (SD). Executive function variables additionally include the observed range interquartile range (IQR).

Abbreviations: BMI, Body Mass Index; BMIz, Body Mass Index *z*‐score; SD, standard deviation; DSB, Digit Span Backwards; SST, Stop signal task; WCST, Wisconsin Card Sorting Test.

### 3.2 Parent and Child Energy Intake and UPFs Consumption

Total mean energy intake was 1689 kcal (SD = 401) for children and 1694 kcal (SD = 504) for parents. Table 2 presents the mean UPFs intake for both parents and children. Total UPFs intake (% of total energy) accounted for a substantial proportion of total energy intake among both parents and children. Among parents, total UPFs intake ranged from 25% to 78% of energy intake (mean = 54.2%, SD = 13.0%, IQR = 19.8; CV = 23.9%) and among children, it ranged from 25% to 90% (mean = 61%, SD = 13%, IQR = 17.4; CV = 21.1%), indicating moderate variability across participants.

**TABLE 2:** Distribution of total ultra‐processed foods (UPFs) intake and UPFs subgroups among parents and children.

Characteristic	Parent					Child				
	Mean (SD)	Range	IQR	CV (%)	Range/SD ratio	Mean (SD)	Range	IQR	CV (%)	Range/SD ratio
Total ultra‐processed foods	54.49 (13.02)	25–78	19.78	23.90	4.12	60.54 (12.70)	25–90	17.44	21.13	5.08
Ultra‐processed savoury snacks	1.70 (2.32)	0–11	2.65	136.52	4.81	2.07 (2.88)	0–14	3.19	138.88	4.77
Ultra‐processed grains	23.38 (10.34)	0–58	13.49	44.25	5.57	26.11 (10.16)	5.7–56	13.99	38.91	4.95
Ultra‐processed meats	3.01 (3.12)	0–15	2.79	103.53	4.75	5.50 (5.95)	0–30	6.15	108.23	5.11
Ultra‐processed dairy	7.21 (6.37)	0–48	5.88	88.33	7.60	10.38 (6.84)	0–44	9.22	65.97	6.46
Ultra‐processed fats	5.67 (3.53)	0–22	3.69	62.28	6.22	4.61 (3.13)	0–15	4.03	67.77	4.79
Ultra‐processed sugars and desserts	8.38 (8.76)	0–41	9.69	104.57	4.70	6.13 (6.10)	0–33	7.23	99.50	5.44
Ultra‐processed sweetened beverages	3.71 (5.32)	0–29	6.0	143.38	5.96	5.05 (6.01)	0–27	7.86	119.13	4.52
Ultra‐processed artificially sweetened beverages	1.06 (1.93)	0–9	1.11	181.66	4.65	0.65 (1.20)	0–7	1.03	183.54	5.64

*Note:* Variables shown as the percentage of energy from the total energy intake.

Abbreviation: SD, standard deviation.

Major UPFs subgroups, including grains, dairy products, sugar‐sweetened desserts and sweetened beverages, also showed broad intake ranges, indicating heterogeneity. In contrast, the subcategory of artificially sweetened beverages showed significant floor effects, with little to no consumption among most participants (see Table 2).

As shown in Table 3, Pearson correlation analyses showed small but statistically significant associations between parent and child food intake, including total energy intake, total UPFs and some UPFs subgroups such as savoury snacks, meats and sweetened beverages. Other UPF subgroups, including grains, dairy, fats, sugars and desserts and artificially sweetened beverages, were not significantly correlated. These associations remained statistically significant in models adjusted for covariates; however, most were weak to modest, except for meats, which showed a stronger association. Parent intake, including energy intake (*β* = 0.15, *p* = 0.024), total UPFs intake (*β* = 0.20, *p* = 0.018), savoury snacks (*β* = 0.28, *p* = 0.018), sweetened beverages (*β* = 0.24, *p* = 0.023) and meats (*β* = 0.60, *p* < 0.001) were associated with child intake.

**TABLE 3:** Associations between parental and child UPFs intake.

Predictor	*r* ^*a*^	*p*	*β*	SE	*t*	*p*
Energy intake (kcal/day)	0.17	0.011	0.15	0.07	2.29	0.024
Total ultra‐processed foods (% e)	0.17	0.036	0.20	0.08	2.40	0.018
Ultra‐processed savoury snacks (% e)	0.21	0.013	0.28	0.12	2.40	0.018
Ultra‐processed grains (% e)	0.10	0.168	0.12	0.09	1.38	0.170
Ultra‐processed meats (% e)	0.18	< 0.001	0.60	0.16	3.67	< 0.001
Ultra‐processed dairy (% e)	0.16	0.066	0.12	0.10	1.22	0.223
Ultra‐processed fats (% e)	0.12	0.128	0.10	0.08	1.28	0.203
Ultra‐processed sugars and desserts (% e)	0.11	0.595	0.01	0.06	0.22	0.824
Ultra‐processed sweetened beverages (% e)	0.13	0.016	0.24	0.11	2.30	0.023
Ultra‐processed artificially sweetened beverages (% e)	0.14	0.351	0.09	0.06	1.63	0.106

Abbreviations: % e, percentage of energy; SD, standard deviation.

^a^ Pearson's correlation coefficients.

### 3.3 Association Between UPFs and EF

**FIGURE 1:**
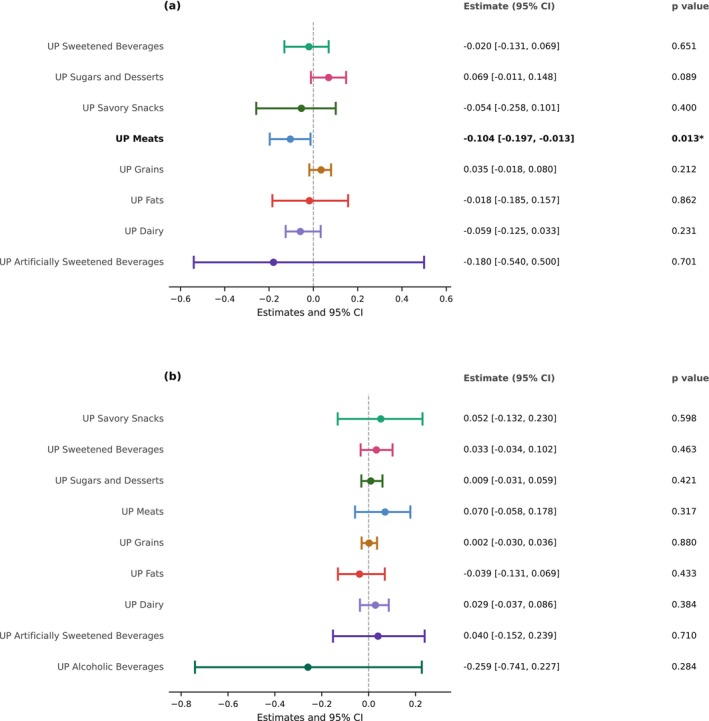
Association of UPFs with working memory among children (a) and parents (b), controlling for covariates. UP, ultra‐processed. Estimates represent standardised regression coefficients with 95% confidence intervals. Bold labels indicate statistically significant associations.

**FIGURE 2:**
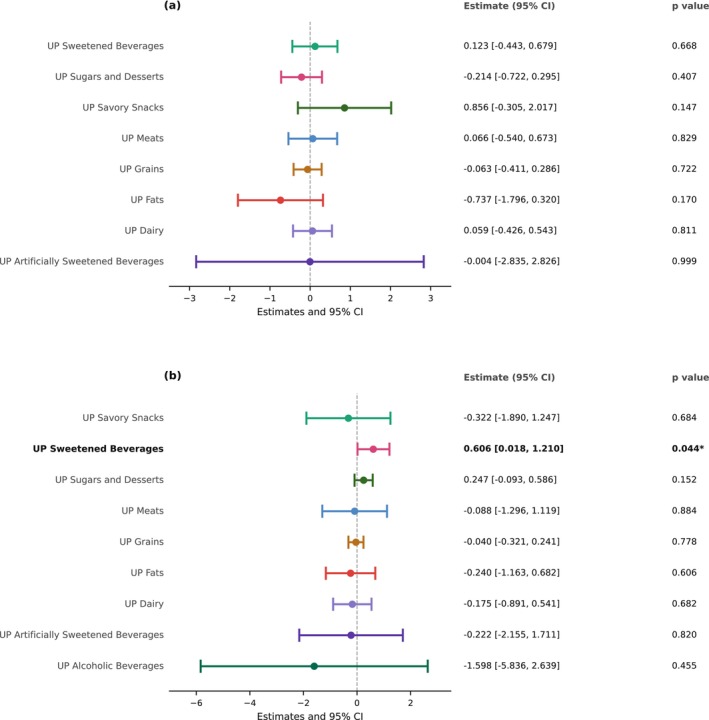
Association of UPFs with inhibitory control among children (a) and parents (b), controlling for covariates. UP, ultra‐processed. Estimates represent standardised regression coefficients with 95% confidence intervals. Bold labels indicate statistically significant associations.

**FIGURE 3:**
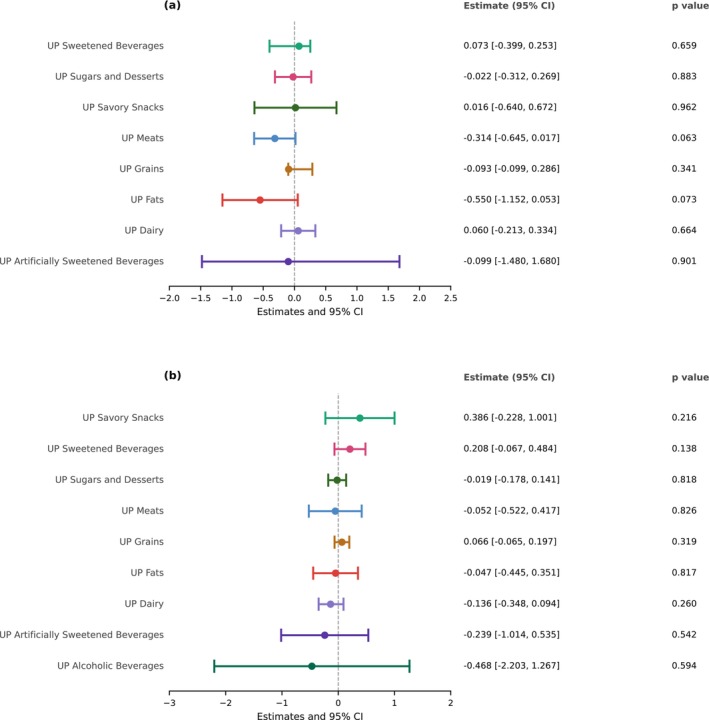
Association of UPFs with cognitive flexibility among children (a) and parents (b), controlling for covariates. UP, ultra‐processed. Estimates represent standardised regression coefficients with 95% confidence intervals. Bold labels indicate statistically significant associations.

Among children, adjusted regression models confirmed a small but statistically significant association between the consumption of ultra‐processed meats (percentage of energy) and lower DSBss (*β* = −0.104 95% CI [−0.197, −0.013], *p* = 0.013) (See Figure 1a). Conversely, no significant associations were observed between UPFs and SST or WCST in adjusted models (See Figures 2a and 3a). No significant differences in associations between UPFs and SST or WCST by child sex were observed (data not shown).

Among parents, there were no significant associations between UPFs intake and DSBss (See Figure 1b). However, the consumption of ultra‐processed sweetened beverages was associated with an elevated percentage of failed trials in the SST (*β* = 0.606, 95% CI [0.018, 1.210], *p* = 0.044) (see Figure 2b). Furthermore, no significant associations were identified between UPFs intake and the WCST among parents in adjusted models (See Figure 3b). Analyses stratified by parent sex were not conducted due to insufficient sample size limiting statistical power.

## 4 Discussion

To our knowledge, this is one of the first studies evaluating the relationship between UPFs and EF in children with obesity and their parents. The findings of this study indicated that UPFs constituted 61% of children's energy intake and 54% of parents' energy intake. This is consistent with other U.S. studies reporting that children consumed a range of 57.7%–67% of energy from UPFs [6], and adults consumed a range of 51%–58% of energy from UPFs [5]. Contrary to our hypotheses, parent–child dietary associations were limited, although small but statistically significant associations were observed for total energy intake, total UPFs, savoury snacks, meats and sweetened beverages. Additionally, most analyses examining UPF subcategories in relation to EF domains were null, suggesting no consistent associations.

Specifically, only higher intake of ultra‐processed meats (e.g., cold cuts, sausages, lean cold cuts, savoury snacks, chicken, fish, meat‐based commercial entrées and fast food) in children was modestly associated with lower working memory scores, while in parents, greater consumption of ultra‐processed sweetened beverages was associated with poorer inhibitory control.

The correlation between child and parent UPFs intake, including total UPFs, savoury snacks, meats and sweetened beverages, was expected, as parents generally handle food shopping and preparation for school‐aged children within the household. These findings are consistent with a recent cross‐sectional study that found a significant association between high UPFs consumption among children and high UPFs consumption among their parents [43]. Similarly, another study reported a positive association between children's intake of processed snacks and their mothers' consumption of processed foods [44].

Consistent with previous research [45], our study found that children consumed a high intake of Ultra‐processed meats, accounting for 5.5% of their total daily energy. Moreover, our analysis suggested that consumption of ultra‐processed meats among children was correlated with diminished working memory capabilities. Ultra‐processed meats encompass a range of food items, including hot dogs, bacon, deli meats, sausages, chicken nuggets, jerky, processed lunch meats and numerous pre‐packaged meat products, all of which are subjected to various industrial processes that increase their fats, SFA, nitrate, sodium, diet‐derived advanced glycation end products (dAGEs) and trimethylamine‐oxide (TMAO) content. These products are recognised for their capacity to impair gut microbiota and influence neuroinflammation [46] and are often found in Western diets. Importantly, these findings suggest an opportunity for intervention, as one study showed that reducing children's consumption of red meat and sausages was related to improved cognition over two years [47]. Given the sensitive neurodevelopmental period of childhood, an increased consumption of ultra‐processed meats during this critical period may exert a more significant impact on their developing brains.

Interestingly, our study found that high consumption of ultra‐processed sweetened beverages was linked to lower parental inhibitory control. This finding is novel, as most prior research evaluating the influence of inhibitory control and sugar‐sweetened beverages has been conducted in children [48]. One study found that consumption of sweetened carbonated beverages decreased cognitive control related to food cues in the prefrontal cortex among adults with obesity [49]. Conversely, another study involving young, healthy adults did not find a relationship between inhibitory control and soda consumption [50]. Moreover, research shows that daily sweetened beverage consumption altered hedonic brain responses [51]. This is noteworthy as low inhibitory control was associated with higher intake of high‐calorie foods [22].

Contrary to our hypothesis, no associations were observed between UPFs and any of the other EF variables in parents or children. This consistent lack of associations may be attributed to several factors. First, the relatively small sample size may have limited statistical power to detect effects across all UPF subcategories. Second, the restricted BMI inclusion criteria may have reduced variability in exposures and outcomes, limiting the ability to detect significant relationships. Third, underreporting of specific UPFs categories due to recall or estimation bias, especially for foods perceived as unhealthy [52], may have weakened the observed associations. Fourth, the EF domains used in this study were designed to measure general cognitive abilities and might be less sensitive to food‐related decision‐making, which also involves emotional and motivational factors that these tasks do not capture. The finding of associations only in specific subgroups suggests differential effects by UPFs subcategories, with some foods potentially exerting more direct physiological influences (e.g., on glucose metabolism, inflammation or vascular function) and others having weaker or more indirect effects. Prior studies with adults have shown that UPFs are associated with a faster decline in overall cognition, working memory and attention [19, 20]. Additionally, food additives and toxic substances resulting from food processing and packaging contribute to disruptions in the endocrine system, oxidative stress and neuroinflammation through their effects on gut microbiota [7]. For example, the phthalates used in UPFs packaging have been associated with detrimental effects on children's cognitive development at the age of six following prenatal exposure, as evidenced in the Ma'anshan birth cohort (MABC) study [53]. Furthermore, dAGEs such as furosine, acrylamide, heterocyclic amines and 5‐hydroxymethylfurfural are linked with inflammation and oxidative stress [54], which have been correlated with accelerated rates of global cognitive decline, episodic memory and perceptual speed deterioration [54]. Another detrimental compound, TMAO, prevalent in ultra‐processed meats, has been associated with cardiovascular disease (CVD) and impairments in learning and memory [46].

### 4.1 Strengths and Limitations

To the best of our knowledge, this is the first study to simultaneously evaluate the relationship between UPFs and EF in children with obesity and their parents, addressing research gaps by examining treatment‐seeking parent–child dyads and providing novel insights into intergenerational dietary patterns and family‐level mechanisms that may inform family‐based paediatric obesity interventions. In this study, we used the NOVA UPFs categorisation, which is considered the gold standard in epidemiological studies [30]. Further strengths of this study included the use of three 24‐h dietary recalls on three non‐consecutive days. Additionally, this research identified specific UPFs products associated with EF impairments, as we calculated not only the total consumption of UPFs but also the different UPFs products consumed by children and their parents. However, there are also limitations to consider.

Given the cross‐sectional design, this study relied on one‐time measurements of treatment‐seeking children with overweight or obesity and their parents, thereby limiting causal interpretation and generalisability to non‐treatment‐seeking or normal‐weight populations. Specifically, by targeting this population, we reduced variability in BMI‐related characteristics and dietary behaviours compared with a population‐based sample, potentially limiting our ability to detect differences. Reduced BMI variability can also attenuate observed associations by narrowing the range over which exposure‐outcome relationships can be detected. Therefore, the present findings should be interpreted cautiously. Although the sample size was sufficient for the primary analyses, it limited our ability to conduct stratified analyses by demographic and socioeconomic characteristics. Larger epidemiological studies are needed to examine subgroup‐specific associations and assess potential effect modification by age, sex and socioeconomic status. Furthermore, the selected EF domains assess general cognitive abilities and may be less sensitive to capturing food‐related decision‐making, which involves processes beyond these tasks. Moreover, dietary recalls are susceptible to biases such as self‐reporting and memory lapses, with studies indicating that individuals with overweight or obesity may underreport or inaccurately report food items [55]. In addition, classifying foods as UPFs with the NOVA system is complex. Research suggests that misclassification using the NOVA system ranges from 5% to 10% [56]. Additionally, other dietary quality approaches are worth exploring, such as the Healthy Eating Index and the Western diet score.

## 5 Conclusion

Dietary intake represents a critical determinant of cognitive function and development, and EF plays a key role in food selection and consumption. This cross‐sectional baseline analysis found limited associations between parent and child UPFs intake across the different categories. Most associations between UPFs and EF domains in children and parents were also null. Our findings showed that consumption of ultra‐processed meats was significantly associated with impaired working memory in children, and consumption of ultra‐processed sweetened beverages was significantly associated with lower inhibitory control in parents. These findings suggest that, while UPFs intake is highly prevalent among adults and children, their relationship with EF should continue to be studied. Future research with broader samples and different study designs, including longitudinal studies, could further examine associations between UPFs and EF among children and their parents and develop parent‐based intervention strategies to reduce UPF consumption and preserve EF health.

## Author Contributions

**Ingrid Rivera‐Iñiguez:** writing – original draft and review and editing, formal data analysis, data curation, methodology, conceptualization. **David R. Strong:** writing – review and editing, data analysis supervision, funding acquisition. **Kyung E. Rhee:** writing – review and editing, funding acquisition. **Dawn M. Eichen:** writing – review and editing. **Kerri N. Boutelle:** writing – review and editing, supervision, methodology, funding acquisition, and conceptualization.

## Funding

This study was supported by the National Institute of Health (NIH) (grant numbers R01DK075861 and K02HL1120242), PI: Boutelle. The content is solely the authors' responsibility and does not necessarily represent the official views of the National Institutes of Health.

## Conflicts of Interest

The authors declare no conflicts of interest.

## Supporting information


**Table S1:** Demographic survey items.
**Table S2:** Food classification according to the NOVA system.

## Data Availability

The UC San Diego IRB (#160289) reviewed and approved the study's written informed consent documents and determined that data sharing according to the language used in the study (2011‐2015) would not be in keeping with the consents signed by subjects and that data sharing would be a violation of the ethical principle of respect for persons. The protocol is available from the primary author.
