# Supplementary material for: Associations of Ultra‐Processed Foods and Executive Function in Children With Obesity and Their Parents: A Cross‐Sectional Study
Source: Pediatr Obes. 2026 Aug 16;21(8):e70143. doi: 10.1111/ijpo.70143 (PMC13478359; doi:10.1111/ijpo.70143)
Supplement: Supplementary file 1 [file IJPO-21-e70143-s001.pdf]

Associations of ultra-processed foods and executive function in children with obesity and their parents: a cross-sectional study

Ingrid Rivera-Iñiguez[1*], David R. Strong[2], Kyung E. Rhee[1], Dawn M. Eichen[1], Kerri Boutelle[1,2,3]

[1] University of California San Diego, Department of Pediatrics, San Diego, CA USA

[2] University of California San Diego, Herbert Wertheim School of Public Health and Human Longevity Science, San Diego, CA USA

[3] University of California San Diego, Department of Psychiatry, San Diego, CA USA

***Corresponding author:** Ingrid Rivera-Iñiguez, Ph.D. University of California San Diego, 9500 Gilman Drive #0874, La Jolla, CA, 92093, USA. iriverainiguez@health.ucsd.edu ORCID: 0000-0001-6129-6528

This supporting information includes: Supplementary Table 1, Demographic Survey Items and Supplementary Table 2, Food classification according to the NOVA system.

SUPPLEMENTARY TABLE 1 | Demographic Survey Items

Socio-demographic Variable	Description / Survey Item
Child ethnicity/race	Parent selects the child's ethnic group(s) (e.g., American Indian/Alaskan Native, Asian, Native Hawaiian/Pacific Islander, Black/African American, White/Caucasian, Other).
Child ethnicity/race	Whether the child is Hispanic/Latino(a) or not
Child ethnic group	American Indian or Alaskan Native, Asian, Native Hawaiian or Other Pacific Islander, Black or African American, White or Caucasian, or other
Child’s age	Age of participating child
Child’s sex	Male or Female
Number of siblings	Number of siblings the child has
Parent sex	Male or Female
Relationship to participating child	Biological mother, adoptive mother, female guardian, biological father, adoptive father, male guardian, other.
Parent age	Age of responding parent.
Parent ethnicity/race	Whether the parent describes themselves as Hispanic/Latino(a) or not
Parent ethnic group	American Indian or Alaskan Native, Asian, Native Hawaiian or Other Pacific Islander, Black or African American, White or Caucasian, or other
Marital status	Single, married, living with partner, divorced, separated, widowed.
Parent employment status	Working full-time, working part-time, homemaker, unemployed/retired/student.
Other adult employment status	Work status of another adult living in the home (if applicable).
Educational attainment	Highest level of education completed by the respondent, the respondent’s mother, the respondent’s father, and the respondent’s spouse/partner. **Options**: Less than 7th grade, Up to 9th grade, Some high school (10th or 11th grade), High school graduate, Some college (at least one year), College graduate, Graduate or professional degree or prefer not to answer
Occupation	Occupational category for respondent, respondent’s parents, and spouse/partner (10-level occupational classification).
Household income	Source of income (one income, two incomes, unemployment, public assistance, child support/alimony, other).
Annual household income	Household income category (e.g., <$9,999 to >$100,000).
Household composition	Presence of another adult in the household.

**SUPPLEMENTARY TABLE 2** | Food classification according to the NOVA system	
**Category 1 Unprocessed or minimally processed foods**	
**Fruits**	Citrus juice home made
	Fruit juice excluding citrus juice home made
	Citrus fruit
	Fruit excluding citrus fruit
	Avocado and similar
**Vegetables**	Dark green vegetables
	Deep yellow vegetables
	Tomato
	White potatoes
	Other starchy vegetables
	Vegetable juice home made
	Legumes (cooked dried beans)
	Other vegetables
	Fried vegetables
**Grains**	Grains flour and dry mixes whole grain
	Grains flour and dry mixes some whole grain
	Grains flour and dry mixes refined grain
	Pasta whole grain
	Pasta some whole grain
**Meats**	Beef
	Lean beef
	Veal
	Lean veal
	Lamb
	Lean lamb
	Fresh pork
	Lean fresh pork
	Game
	Poultry
	Lean poultry
	Fish fresh and smoked
	Lean fish fresh and smoked
	Shellfish
	Organ meats
	Eggs
	Meat alternatives
**Dairy**	Milk whole
	Milk reduced fat
	Milk low fat and fat free

**Beverages**	Unsweetened tea
	Unsweetened coffee
	Unsweetened water
**Category 2. Processed culinary ingredients**	
**Vegetables**	Pickled Vegetables
**Dairy**	Milk nondairy
**Sugar**	Sugar
**Fats**	Cream
	Cream reduced fat
	Cream low fat and fat free
	Cream nondairy
	Oil
	Butter and other animal fats regular
	Butter and other animal fats reduced fat
	Non grain flour and similar
	Nuts and seeds
	Nuts and seeds butters
**Category 3. Processed foods**	
**Fruits**	Fried fruits
	Citrus juice sweetened
	Fruit juice excluding citrus juice sweetened
	Fruit based savory snack
**Vegetables**	Vegetable juice packaged
**Grains**	Popcorn
	Loaf type bread and plain rolls whole grain
	Loaf type bread and plain rolls some whole grain
	Other breads (quick breads, corn muffins, tortillas) whole grain
	Other breads (quick breads, corn muffins, tortillas) some whole grain
	Crackers whole grain
	Crackers some whole grain
	Ready-to-eat cereal (not presweetened) whole grain
	Ready-to-eat cereal (not presweetened) some whole grain
	Ready-to-eat cereal (presweetened) whole grain
	Ready-to-eat cereal (presweetened) some whole grain
	Cakes, cookies, pies, pastries, Danish, doughnuts and cobblers wholegrain
	Cakes, cookies pies, pastries, Danish, doughnuts, and cobblers some wholegrain
	Pasta refined grain
	Snack bars whole grain

	Snack bars some whole grain
	Snack chips whole grain
	Snack chips some whole grain
**Category 4. Ultra-processed foods and drinks**	
**Ultra-processed Savory snacks**	Fried potato chips
	Vegetable-based savory snacks
**Grains**	Loaf type bread and plain rolls refined grain
	Other breads (quick breads, corn muffins, tortillas) refined grain
	Crackers refined grain
	Ready-to-eat cereal (not presweetened) refined grain
	Ready-to-eat cereal (presweetened) refined grain
	Cakes, cookies, pies, pastries, Danish, doughnuts and cobblers refined grain
	Snack bars refined grain
	Snack chips refined grain
	Flavored popcorn
**Ultra-processed Meats**	Cured pork
	Egg substitute
	Lean cured pork
	Fried chicken commercial entrée and fast-food
	Fried fish commercial entrée and fast-food
	Fried shellfish commercial entrée and fast-food
	Cold cuts and sausage
	Lean cold cuts and sausage
	Meat based Savory snack
**Ultra-processed Dairy**	Ready-to-drink flavored milk whole
	Ready-to-drink flavored milk reduced fat
	Ready-to-drink flavored milk low fat and fat free
	Sweetened flavored milk beverage powder with nonfat dry milk
	Artificially sweetened flavored milk beverage powder with nonfat dry milk
	Sweetened flavored milk beverage powder without nonfat dry milk
	Artificially sweetened flavored milk beverage powder without nonfat dry milk
	Cheese full fat
	Cheese reduced fat
	Cheese low fat and fat free
	Cheese nondairy
	Yogurt sweetened whole milk
	Yogurt sweetened low fat

	Yogurt sweetened fat free
	Yogurt artificially sweetened whole milk
	Yogurt artificially sweetened low fat
	Yogurt artificially sweetened fat free
	Yogurt nondairy
	Frozen dairy dessert
	Frozen nondairy dessert
	Pudding and other dairy dessert
	Artificially sweetened pudding and other dairy dessert
**Ultra-processed Fats**	Margarine regular
	Margarine reduced fat
	Shortening
	Salad dressing regular
	Salad dressing reduced fat
	Gravy regular
	Gravy reduced fat and fat free
	Sauces and condiments regular
	Sauces and condiments reduced fat
	Soup broth
**Ultra-processed Sugar and Desserts**	Miscellaneous dessert
	Sugar substitute
	Syrup honey and jam
	Sauces sweet regular
	Sauces sweet, reduced fat, reduced calorie or fat free
	Chocolate candy
	Non chocolate candy
	Frosting or glaze
**Ultra-processed Sweetened Beverages**	Sweetened soft drinks
	Sweetened fruit drinks
	Sweetened tea
	Sweetened coffee
	Sweetened coffee substitutes
	Sweetened water
**Ultra-processed Unsweetened Beverages**	Unsweetened soft drinks
	Unsweetened coffee substitutes
**Ultra-processed Artificially**	Artificially sweetened fruit drinks
	Artificially sweetened tea
	Artificially sweetened coffee
	Artificially sweetened coffee substitutes

**sweetened Beverages**	Artificially sweetened water
	Artificially sweetened soft drinks
**Ultra-processed Alcoholic Beverages**	Beer and ales
	Cordial and liqueur
	Distilled liquor
	Wine
